# The Economics of Dialysis in India

**DOI:** 10.4103/0971-4065.50671

**Published:** 2009-01

**Authors:** Umesh Khanna

**Affiliations:** Mumbai Kidney Foundation, Mumbai, India

Chronic kidney disease is a worldwide public health problem, a social calamity and an economic catastrophe. In the year 2000, in the United States (US) alone, about 30 million people were diagnosed with chronic kidney disease (CKD). It is estimated that by 2010, six million worldwide would need renal replacement therapy (RRT) costing 28 billion dollar.[1]

## Burden of CKD in India

The exact burden of CKD in India still remains undefined with only limited data from the three population-based studies addressing this issue.[2–4]

It is hoped that the CKD registry, recently established by the Indian Society of Nephrology, may provide useful epidemiological data in the future. In the prevention study done in Chennai, the prevalence at the community level is 8600 per million population (pmp) in the study group and 13900 pmp in the control group. The second study based in Delhi[3] revealed a prevalence of CKD (serum creatinine more than 1.8 mg %) at 7852 pmp. The third study from Bhopal revealed an incidence of 151 pmp suffering from end stage renal disease (ESRD). Do we have the resources and skill to handle this ever increasing population of ESRD in India?

## Economic Scenario in India

As per the December 2007 index declared by Rajya Sabha, the per capita income in India is Rs 20734 per annum. The total population is 113 crore of which 26% live below the poverty line (BPL) where the daily earning is Rs 10, in comparison, the international standard BPL is US $1 per day i.e. Rs 45 per day. By this parameter, in India, 70% of the population would be BPL. The government spends barely US $8 per capita on health with priorities more on infectious disease, sanitation, nutrition etc[4] (MK Mani *et al*.)

## Facilities for RRT in India

In the absence of any available data, Mumbai Kidney Foundation (MKF) conducted a data collection exercise with the help of industry, sources, personal discussion with nephrologists and telephonic confirmation of dialysis centers.

India has close to 950 nephrologists (not all ISN members) all over the country. There are 700 dialysis centers with a total of 4000 dialysis machines, predominantly in the private sector and mainly concentrated in cities, especially metros. There are around 20,000 patients undergoing dialysis at these centers [Tables 1 and 2]. There are around 170 government recognized transplant centers in India, performing around 3500 transplants annually. The patients on CAPD number less than 5000. Clearly, the choices and facilities for RRT are predominantly focused on maintenance hemodialysis and are woefully inadequate.

**Table 1:** State of RRT zone wise

Zone	No of Dialysis centers	No. of Dialysis Machines	No. of dialysis per month	Cost of dialysis per session	No. of transplant centers	No. of transplant per month
North	229	1106	50,560	1250	26	85
South	306	1453	85,440	1100	88	117
East	108	430	27,050	1350	15	25
West	175	1000	90,000	1000	37	71

**Table 2:** State of RRT city wise

City	No of Dialysis centers	No. of Dialysis Machines	No. of dialysis per month	Cost of dialysis per session	No. of transplant centers	No. of transplant per month
Delhi	79	490	28,500	1600	10	35
Mumbai	112	600	40,000	750	20	16
Chennai	44	146	10,220	1200	17	34
Calcutta	36	250	20,000	1100	10	20

## Cost of RRT in India

The MKF data also gave insights into the costing of ESRD management [Table 3].

**Table 3:** Zone and city wise cost of dialysis in India

Zone	Cost of dialysis (Rs) per session
North	1250
South	1100
East	1350
West	1000
Delhi	1600
Mumbai	750
Chennai	1200
Calcutta	1100

The cost of each hemodialysis (HD) session in India varies from Rs 150 in government hospitals to Rs 2000 in some corporate hospitals. The monthly cost of HD in most private hospitals average Rs 12000 and the yearly cost of dialysis is Rs 1, 40000, equivalent of $3000, which is in sharp contrast to an annual cost of $60,000 in the US and UK. So we are the cheapest in the world and yet more than 90% of Indians cannot afford it.

The cost of an AV fistula construction is Rs 6000 to Rs 20000 from a government hospital to varying grades of private hospitals. The average cost of erythropoietin per month is Rs 4000 (bio similar) to Rs 10000 (the pioneer brand).

The average cost of kidney transplant varies from Rs 50000 in a government set-up to Rs 300000 in an average private hospital. Also the yearly maintenance cost post transplant for drugs amounts to Rs 12 0000 per year or Rs 10000 per month.

## Actual Cost Break-up of Dialysis in a Private set up

MKF conducted a survey of actual costing of dialysis in few private hospitals in different cities of India [Table 4] and asked for their running cost of maintenance of hemodialysis in centers that have a chronic dialysis program and we came up with interesting findings.

**Table 4:** Actual costing of Dialysis

Consumables	Rs 350
Electricity	Rs 50
Water, telephone, Insurance	Rs10
Annual Maintenance - dialysis machine RO plant, premises	Rs 20
Repairs & wear & tear	Rs 20
Depreciation	Rs 100
Honorarium to nephrologist	Rs 100-200
Staff Salary	Rs 100-200
Total	Rs 750-950

# Interest on capital Expenditure - Variable

- the cost differed from a nephrologist owned facility versus a corporate hospital
- the administration greatly exaggerated the cost
- Even in large corporate hospitals the recurrent cost dialysis worked out to be between Rs 700-900 as shown in the Table 4.

The average cost to the patient across the country works out to be Rs 1100, which truly is, beyond the reach of more than 90% of India. To increase the reach of dialysis, bring it out of the corporate hospital set up and make it cheaper in the smaller free standing units or nephrologists-owned units.

## Concept of Nephrologist Owned Unit

Is it cost effective? The answer is yes! If you do not mind the travails of running a center. One could either own the set up or take it on lease and pay rent. It could be a day care centre attached to your consulting room, or a full fledged small hospital with indoor facilities.

By my own experience, the cost of a single dialysis session to the nephrologist comes to less than 600 if we make bulk purchases. Laboratory facilities or outsourcing can fetch additional income. Sale of EPO after obtaining a pharmacy license is easy and lucrative and can easily subsidize the cost of dialysis.

## Our Experience in Mumbai

If you go through the table, you will realize that Mumbai has the distinction of offering the cheapest dialysis in India at an average cost of Rs 700 per session largely because of the presence of two distinct models which have been in existence since many years [Table 5].

**Table 5:** Costing of our 700 dialysis done last month in a nephrologist-owned unit

Items	Monthly Expenses (Rs.)	Per dialysis (Rs.)
Formalin	1470	2
Hypochloride	630	1
Hydrogen Peroxide	480	0.7
Acitic Acid	1110	1.6
Dialyzers (118 no)	60,000	86
Bicarb Cans (303 can)	45,000	64
Tubing Set (32 sets)	4,160	6
A-V.Fistula Needles (1400)	29,400	42
Normal saline (1 Liter 707 bottles	14,140	20
Ns 500 Ml 350	4,550	6.5
Gloves (Per day 2box × 26 Days)	9,256	13
Heparine (250 Vials Of 25000 Units)	7,000	10
Gauze (40packs)	10,080	14.4
Syringes (20ml 707)	5,567	8
Micropore Sticking Plaster	1,616	2.3
Neosporin Powder	2,418	3.5
IV Set (707 pieces)	5,656	8
Material Total expenses	202533	289

## Concept of Free Standing Unit

Also called community dialysis centre or satellite dialysis units, they essentially offer only dialysis facility with no admission facility. Thus, they can offer dialysis at a reasonable price by cutting down the overheads. It comprises of a full fledged dialysis centre with 10-20 dialysis machines, isolated machines for Hepatitis B and C, RO plant and a resuscitation trolley with monitor and defibrillator. It also has trained nurses, technicians and doctors trained in resuscitation of a serious patient. The patient is screened at the entry point and taken only if he is stable for OPD dialysis. Semi-acute problems are solved by telephonic consultation between the RMO and nephrologist. During an occasional acute emergency, the RMO or the paramedics resuscitate and if need be transfer to a hospital.

The nephrologist reviews the patient at least once in a week, if not daily. Such a concept of free standing unit already exists in the USA and Singapore. Based on this model, presently, Mumbai has 17 such free standing units out of a total of 112 dialysis centers in the city.

## Concept of Charitable Dialysis Unit

The second model, which brought down the cost of dialysis further down in Mumbai, was the involvement of philanthropists and setting up of non-governmental organization (NGO) backed charitable dialysis units, either in an established hospital or as free standing units.

The involvement of the NGO can be in the following manner.

- The entire space and machinery belongs to the trust and they provide dialysis on a no profit no loss basis.
- The NGO is interested only in donating machines and RO plant to an already existing private dialysis centre and in lieu of the donation gets a fixed number of free dialysis to help poor patients.
- The philanthropist is interested in donating dialysis machine and wants the unit to be named after him or gets into the advisory board of the dialysis unit.
- The NGO enters into a public-private partnership with the government which provides space for the unit in a government hospital and the NGO runs the charitable dialysis unit.

Other areas in which NGOs or philanthropists work in dialysis field are:

- Bulk purchase of medicines especially injection Erythropoietin and providing them at an extremely subsidized price to dialysis patients.
- Bulk purchase of dialyzers, tubings and other disposable items used for dialysis and selling them to patients at subsidized rates.
- Offering pick up and drop services to dialysis patients.
- Offering free monthly rations to poor CKD patients.
- Holding blood donation camps for providing blood to dialysis patients.

As a result of promoting these two concepts, Mumbai has 112 dialysis centers, of which 17 are free standing units. Of the 600 dialysis machines which Mumbai has, 150 are in charitable units. In Mumbai, there are about 5000 dialysis patients of which 1250 are taking dialysis at less than Rs 350 per dialysis and the average price of dialysis is Rs 700 - 750 per session, which is the cheapest in the country (National average cost is Rs 1100). Further more, some patients pay less than Rs 100 per dialysis.

## Suggestions for Increasing the Outreach of Dialysis all over India

In summary what can be done to make dialysis more affordable?

The Mumbai model can be used as an example to improve the availability as well as affordability of dialysis therapy all over the country. This can be done in the following manner:

- Build middle level cost effective nephrologist-owned dialysis units costing Rs 800 per session as dialysis cost.
- Encourage free standing units or satellite dialysis units
- Involve philanthropists to adopt patients and donate machines. There is no dearth of such people wanting to help for a good cause.
- Encourage the govt. to lend space for private public partnership in a govt. hospital with NGO participation.
- Lobby with the govt. to subsidize or make dialysis equipment and disposables tax free. *If multiplexes can enjoy tax free holidays,* dialysis units also deserve some help from the government. Also, let the electricity used by such units be charged at non-commercial rates.
- Buy cheap labor by training intelligent, smart, non-graduates to become dialysis technicians, thereby bringing down the labor cost. *ISN can start such courses.*
- ISN should back its nephrologists for every medico legal case arising out of there subsidized dialysis units. This can be done by laying minimum standard of care criteria which the units need to adhere to

Thus one can be cynical and say there are two options - let patients succumb to their illness as Indians cannot afford RRT or adopt the Mumbai model of providing RRT at highly subsidized rates.
